# Database of non-target invertebrates recorded in field experiments of genetically engineered Bt maize and corresponding non-Bt maize

**DOI:** 10.1186/s13104-022-06021-3

**Published:** 2022-06-06

**Authors:** Michael Meissle, Steven E. Naranjo, Jörg Romeis

**Affiliations:** 1 Agroscope, Research Division Agroecology and Environment, Reckenholzstrasse 191, 8046 Zurich, Switzerland; 2 USDA-ARS, Arid-Land Agricultural Research Center, 21881 North Cardon Lane, Maricopa, Arizona, 85138 USA

**Keywords:** *Bacillus thuringiensis*, Corn, Critical appraisal, Cry protein, Database, Environmental risk assessment, Genetic engineering, Meta-analysis, Non-target organisms, Systematic review

## Abstract

**Objectives:**

To assess potential non-target effects of genetically engineered/modified (GM) maize that produces insecticidal proteins from *Bacillus thuringiensis* (Bt), numerous field experiments have been conducted worldwide. Field data are often variable and influenced by uncontrolled factors and meta-analyses can recognize general effects with increased statistical power compared to individual studies. This database represents a comprehensive collection of experimental field data on non-target invertebrates in Bt and non-Bt maize. It was created for a systematic review with the question if growing Bt maize changes abundance or ecological function of non-target animals compared to growing of non-GM maize. Systematic literature searches identified relevant data. Authors were contacted for additional information or raw data if needed and a critical appraisal scheme was developed and applied to each data record.

**Data description:**

The database contains 7279 records of non-target invertebrate abundance, activity density, or predation or parasitism extracted from 120 articles. Records for individual species and life stages, but also aggregated data are available. Each record represents a comparison of invertebrates in Bt and non-Bt maize and includes means, standard deviations and sample sizes. Additional variables characterize publication details, experimental setup, cultivars, Bt proteins, geographic location, field management, insecticide treatments, sampling details, and taxonomy.

## Objective

To assess potential non-target effects of genetically engineered/modified (GM) maize that produces insecticidal proteins from *Bacillus thuringiensis* (Bt) compared with non-Bt maize, numerous field experiments have been conducted worldwide over the past 25 years. Field data are often variable and influenced by numerous uncontrolled factors. Meta-analyses have the potential to increase statistical power compared with individual studies, so that general effects over multiple years and locations can be identified. The body of literature has been growing rapidly and a comprehensive, publicly available, and up-to-date database on non-target field data is lacking. The database presented here builds on previous meta-analyses on non-target invertebrate field studies with Bt crops [1–3]. It was created for a systematic review with the question: “Does the growing Bt maize change abundance or ecological function of non-target animals compared to the growing of non-GM maize?” [4, 5] The review was conducted within the EU project GRACE (GMO Risk Assessment and Communication of Evidence) [6]. It is limited to Bt maize because the most non-target field data are available for this widely grown crop. The systematic review followed the guidelines of the Collaboration for Environmental Evidence [7]. Literature published until August 2019 was searched systematically in multiple bibliographic databases, websites, and reference sections of reviews with defined search terms to identify relevant data. References were screened according to a set of inclusion criteria. Authors were contacted for additional information or raw data if the published information was insufficient. A critical appraisal scheme for non-target field data of GM crops was developed and applied to each record in the database. The database has been used as a basis for a range of meta-analyses, including analyses on different non-target taxa and functional groups, different types of Bt maize (Coleoptera-active, Lepidoptera-active, or stacks), and different insecticide treatments (in particular pyrethroids) [5].

## Data description

The database contains records of non-target invertebrates collected within Bt and non-Bt maize fields or plots (Table 1, data file 1 [8]). Data were derived from replicated field experiments. The measured outcome was either abundance, activity density, or predation or parasitism rate. Each record represents a comparison between Bt and non-Bt plots or fields and contains the seasonal means of a particular taxon with a measure of variation (SD) and sample size (N) for each type of maize. Comparisons involved Bt vs. non-Bt maize either untreated or treated with the same insecticides, and untreated Bt maize vs. insecticide-treated non-Bt maize.

**Table 1:** Overview of data files/data sets

Label	Name of data file/data set	File types (file extension)	Data repository and identifier (DOI or accession number)
Data file 1	Database of non-target invertebrates in Bt and non-Bt maize	xlsx file (Microsoft Excel 2016)	Dryad (10.5061/dryad.3j9kd51jq) [8]
Data file 2	List and definitions of variables in the database	docx file (Microsoft Word 2016)	Dryad (10.5061/dryad.3j9kd51jq) [8]
Data file 3	Critical appraisal questions and answer options	docx file (Microsoft Word 2016)	Zenodo (10.5281/zenodo.6517033) [9]

In addition to the quantitative response data, a range of descriptive variables characterize each record (Table 1, data file 2 [8]). Publication details include the source of data, peer-review status, authors, affiliations and funding. Experiments (data from one location and one year) are described by the cultivar and Bt proteins, geographic information, experimental design, field management, and insecticide treatments. Details on the sampling method and interval as well as on the recorded taxon, life stage, and functional group are provided. Further information on data extraction, calculations, and the response variable is given.

In many cases, multiple records are available that represent different taxonomic levels or different life stages of a taxon. For example, records on species level as well as aggregated records on family or order level have been created. Means generally represent seasonal means of one year and the SDs are based on the number of replicated plots or fields. If data were not available in the desired format, approximations were used whenever possible (e.g., averaged means and SDs over individual sampling dates to obtain a seasonal mean). In some experiments multiple Bt maize lines were compared to the same non-Bt control. In those cases, the database contains separate records for each Bt line while the data for the non-Bt line are used repeatedly.

Critical appraisal was applied to each record in the database (Table 1, data file 3 [8]). The different appraisal criteria include 16 questions to assess both internal validity (risk of bias) and external validity (the degree to which the records are appropriate or applicable for answering the review question). Three answer categories were defined: low, medium, and high validity. When information was unavailable, the record was flagged “unreported”. For each criterion, a decision was made if unreported information should be treated as low, medium, or high for the selection of records for meta-analyses, depending on the likelihood that the lack of information reduces validity. For each question, clear cut off values were defined to ensure transparency, consistency, and reproducibility of the judgement.

The database contains 7279 records from 233 experiments and 120 articles (Table 1, data file 1 [8]). The field experiments were performed between 1994 and 2017. In 61% of the records in the database, invertebrates were recorded in Bt and non-Bt maize without insecticide treatment and in 8% of the records, Bt and non-Bt maize received the same insecticide treatment. In 31% of the records invertebrates in untreated Bt maize were compared to those in insecticide-treated non-Bt maize.

## Limitations

For some experiments that were identified in the systematic literature search, data suitable for inclusion into the database could not be obtained, despite the effort of contacting authors [5]. Data not fitting the requirements of the database are summarized in [5]. Shortcomings of the datasets that were entered in the database are principally addressed in the critical appraisal. Critical appraisal questions and cut-off values were specifically designed for this database, because there was no commonly agreed critical appraisal scheme for arthropods collected in GM crop field studies. The three most common issues identified in the critical appraisal were (1) lack of confirmation of Bt protein expression in the plants (57% of records); (2) seasonal mean was based on only three or fewer sampling dates (30% of records); and (3) SD values of seasonal means had to be estimated or recalculated, which introduced uncertainty (18% of records). Furthermore, it has to be noted that the records in the database do not necessarily represent independent observations. Data from control plots were used multiple times if different Bt maize lines in one experiment had the same non-Bt control. Records on different taxonomic levels (e.g., species and family or order), on different life stages (e.g., larvae or adults and all stages combined), and on different sampling methods that may have recorded the same population of arthropods (e.g., visual observations and sweep nets) were also included. If this database is used for meta-analyses, it is thus warranted to select the most appropriate records for the specific statistical model to ensure independence of the analysed data. For the systematic review on non-target effects of Bt maize, such a selection was done [5].

## Data Availability

The data described in this Data note can be freely and openly accessed on Dryad under https://doi.org/10.5061/dryad.3j9kd51jq [8]. Please see Table 1 for details.

## Abbreviations

Bt: *Bacillus thuringiensis*
GM: Genetic modification
SD: Standard deviation
EU: European Union
